# Validation of the juvenile artritis disease activity score in 1124 patient visits

**DOI:** 10.1186/1546-0096-9-S1-P154

**Published:** 2011-09-14

**Authors:** LD de Vries, MW Heijstek, N Groot, M Bulatović, NM Wulffaat

**Affiliations:** 1 Department of Pediatric Immunology, Wilhelmina Children’s Hospital, University Medical Centre Utrecht, The Netherlands

## Aim

To analyze the validity and feasibility of the Juvenile Arthritis Disease Activity Score [1] (JADAS)-27 in patients with juvenile idiopathic arthritis (JIA).

## Methods

1124 visits in 255 JIA patients were evaluated. Correlations of JADAS-27 (range 0-57) with JADAS-10, JADAS-71, core-set criteria, Disease Activity Index (DAS-28), and Clinical Disease Activity Index (CDAI) were analyzed in the total group and treatment groups.

Responsiveness to minimal clinically important difference (MCID) was analyzed longitudinally. Worsening was defined as a flare with at least 20% PGA and 15% ESR increase, as well as starting therapy, or drug dosage escalation. Improvement was defined as reaching ACRped50 or as reduction of medication.

## Results

The distribution of JADAS-27 (median=2.9; range 0-40) was skewed to the left. The JADAS-27 score could not be calculated in 261 visits (23%), due to missing parent/patient global assessment of well-being (n=119) or missing ESR (n=157). The JADAS-27 correlated highly with JADAS-71 (R=1.00), JADAS-10 (R=1.00) and CDAI (R=0.98), moderately with core set criteria (R=0.65 to 0.85) and DAS-28 (R=0.63), and poorly with ESR (R=0.30). JADAS-27 was higher in patients using MTX (median=4.2; p<0.001), and in patients prior to starting anti TNF-alpha treatment (median=13.8; p<0.001).

The minimal clinically important improvement was represented by a median change in JADAS of -5.2 (IQR= 6.6), and deterioration by +3.4 (IQR= 4.3). Improvement was detected in 95% and worsening in 74% of visits.

## Conclusion

The JADAS-27 is a reliable tool for assessing JIA disease activity given its high correlation with other disease activity parameters, its markedly higher scores in patients requiring biologicals, and its ability to detect MCID in the majority of patients**.** However, the high frequency of missing values in the criteria that are needed to calculate the JADAS-27, may hamper its feasibility.
